# Hobnail variant of papillary thyroid carcinoma: molecular profiling and comparison to classical papillary thyroid carcinoma, poorly differentiated thyroid carcinoma and anaplastic thyroid carcinoma

**DOI:** 10.18632/oncotarget.15786

**Published:** 2017-02-28

**Authors:** Lianghong Teng, Wanglong Deng, Junliang Lu, Jing Zhang, Xinyu Ren, Huanli Duan, Shannon Chuai, Feidie Duan, Wei Gao, Tao Lu, Huanwen Wu, Zhiyong Liang

**Affiliations:** ^1^ Department of Pathology, Peking Union Medical College Hospital, Chinese Academy of Medical Science, Beijing, China; ^2^ Department of Pathology, Xuanwu Hospital, Capital Medical University, Beijing, China; ^3^ Burning Rock Biotech Co. Ltd., Guangzhou, China

**Keywords:** papillary thyroid carcinoma, hobnail variant, next-generation sequencing, molecular features, TERT promoter mutation

## Abstract

**Background:**

As a rare but aggressive papillary thyroid carcinoma (PTC) variant, the genetic changes of hobnail variant of PTC (HVPTC) are still unclear.

**Results:**

The prevalence of HVPTC was 1.69% (18/1062) of all PTC diagnosed in our cohort. 73 samples from 55 patients (17 HVPTC, 26 CPTC, 7 PDTC and 5 ATC) were successfully analyzed using targeted NGS with an 18-gene panel. Thirty-seven mutation variant types were identified among 11 genes. BRAF V600E mutation was the most common mutation, which is present in almost all HVPTC samples (16/17, 94%), most CPTC samples (20/26, 77%), and none of the ATC and PDTC samples. TERT promoter mutation (C228T) was identified in 2 ATC and one HVPTC patient. RAS and TP53 mutation are almost exclusively present among ATC and PDTC samples although TP53 mutation was also observed in 3 HVPTC patients. Six different GNAS mutations were identified among 8 CPTC patients (31%) and none of the HVPTC patients. The only patient who died of disease progression harbored concomitant TERT C228T mutation, BRAF V600E mutation and TP53 mutation.

**Methods:**

HVPTC cases were identified from a group of 1062 consecutive surgical specimens diagnosed as PTC between 2000 and 2010. Targeted next-generation sequencing (NGS) was applied to investigate the mutation spectrum of HVPTC, compared to classical PTC (CPTC), poorly differentiated thyroid carcinoma (PDTC) and anaplastic thyroid carcinoma (ATC).

**Conclusion:**

As an aggressive variant of PTC, HVPTC has relatively specific molecular features, which is somewhat different from both CPTC and ATC/PDTC and may underlie its relatively aggressive behavior.

## INTRODUCTION

Incidence of thyroid cancer has increased all over the world in recent decades, primarily due to the increased prevalence of papillary thyroid carcinoma(PTC). In China, thyroid cancer has become the eighth most common malignant tumor in women based on the data of the National Central Cancer Registry (NCCR) in 2015 [1]. Some of the histological variants of PTC, such as tall cell, diffuse sclerosing, and columnar variants, associate with a poor outcome, even though the overall survival rate of PTC at 10 years is greater than 90%. Hobnail variant of papillary thyroid carcinoma (HVPTC) was first described as a new aggressive variant in 2009 characterized by tumor cells harboring hobnail features [2]. Thus far, only a few small cohorts of HVPTC have been reported, with patients from the United States [3–5], Mexico [6], Italy [7] and Korea [8]. All the studies have suggested that HVPTC behave more aggressively than classical PTC (CPTC). Recently, one study reported that the hobnail variant was observed in more aggressive thyroid tumors, such as poorly differentiated thyroid carcinomas, and suggested that hobnail features may be an indication of higher-grade transformation [4]. Although high BRAF mutation frequency (40-80%) has been reported in these studies, this genetic variant alone might not fully account for the higher rate of recurrence and mortality due to the small sample size and limited candidate genes.

Next-generation sequencing(NGS) technology is able to provide simultaneous screening of a variety of genomic aberrations such as single-nucleotide variants (SNVs), multiple-nucleotide variants (MNVs), small and large insertions and deletions, and copy number variation (CNVs) [9, 10]. More importantly, screening multiple markers with NGS technology requires lower input of nucleic acids in contrast to traditional sequencing technologies, which makes NGS very desirable for routine molecular profiling in cancer. NGS has been used in thyroid cytology to improve diagnostic accuracy, especially in indeterminate specimens [11, 12]. Meanwhile, one study also investigated the molecular profiling of the major types of thyroid cancer using the high-throughput NGS panel (ThyroSeq) [13]. Based on this study, point mutations were identified in 30-83% of specific thyroid cancer types, and among PTC, the genetic profiles of follicular variant PTC are dominated by RAS mutation while the BRAF is the major mutation in CPTC. In this study, we applied targeted NGS panel to investigate the mutation spectrum of HVPTC, compared to CPTC, poorly differentiated thyroid carcinoma (PDTC) and anaplastic thyroid carcinoma (ATC).

## RESULTS

### Clinicopathological characteristics

**Figure 1:**
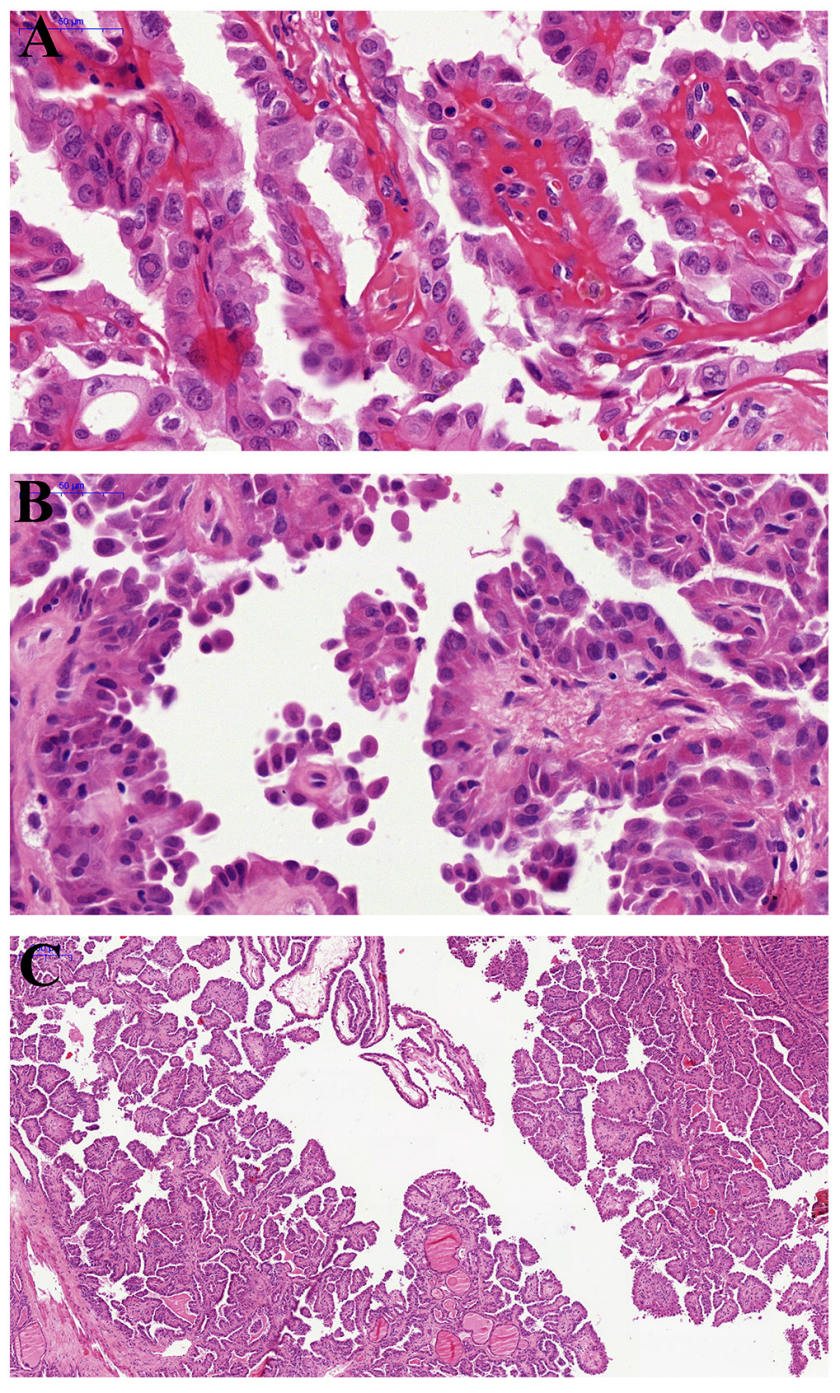
Pathological findings of hobnail variant papillary thyroid carcinoma **(A)** The papillary architecture and characteristic nuclear features of papillary carcinoma: pseudoinclusions and nuclear grooves. **(B)** Micropapillary structures lined by cuboidal cells with apically placed nuclei (“hobnail”appearance) and loss of cellular cohesion. **(C)** The papillary architecture with focal micropapillary areas at low magnification

Table 1 shows the clinicopathological features of the HVPTC. The prevalence of HVPTC was 1.69% (18/1062) of all PTC diagnosed in our institution during the period of 2000-2010. The mean age was 41.8 years (range23-78years) with a clear female predominace (female/male ratio, 13:5). The mean tumor size was 2.53 cm (range1-5cm), with 3 being multifocal (16.7%). Morphologically, papillary and micropapillary structures and hobnail features were identified in all the 18 selected cases (Figure 1A, 1B and 1C). Focal (usually <10%) hobnail appearance was not unusual in some PTC cases, presented on the top of a papilla and the marginal area of the lesion, but we did not put these cases into this cohort due to the selected criteria that hobnail features must be observed in≥30% tumor cells. Extra-thyroidal extension and lymph-vascular invasion were observed in 6 (33.3%) and 2 (11.1%) patients, respectively, whereas lymph node metastasis was identified in 10 patients (58.8%). Hobnail features were not observed in any of our 12 ATC/PDTC cases.

**Table 1:** The clinical and pathologic features of 18 HVPTC cases

Case	Age/sex	Size (cm)	Multifocal	Percentage of hobnail features	Percentage of other variants	LVI	ETE	LN METS	Postsurgical I^131^treatment	Local recurrence	Distant METS	pTNM	Follow up (months)
1	32/M	1.5	No	40%	60%CPTC	No	No	Yes(2/2)	No	No	No	PT_1b_N_1_M_0_	not available
2	76/F	5	No	50%	20%CPTC 10%CCVPTC 10%TCVPTC	Yes	Yes	Not available	No	Yes	Yes(Lung)	PT_3_N_X_M_1_	17m, DOD
3	31/M	1.8	No	100%		No	No	Yes(9/27)	Yes	No	No	PT_1b_N_1_M_0_	100m, NED
4	51/F	2.5	No	80%	10%CPTC 10%DSVPTC	No	Yes	N	Yes	No	No	PT_3_N_0_M_0_	95m, NED
5	25/F	2.2	No	50%	30%CCVPTC 20%CPTC	No	No	N	No	No	No	PT_2_N_0_M_0_	101, NED
6	33/F	1.5	No	80%	20%CPTC	No	No	N	No	No	No	PT_1b_N_0_M_0_	92m, NED
7	49/M	1.6	No	60%	40%CPTC	No	Yes	N	Yes	No	No	PT_3_N_0_M_0_	89m, NED
8	56/F	3.3	No	100%		No	No	Yes(2/7)	Yes	No	No	PT_2_N_1_M_0_	82m, NED
9	38/F	2.5	No	60%	40%CPTC	No	Yes	N	No	No	No	PT_3_N_0_M_0_	74m, NED
10	25/F	2	No	30%	70%CPTC	No	Yes	N	No	No	No	PT_3_N_0_M_0_	82m, NED
11	27/F	3.1	No	80%	20%CPTC	No	No	N	No	No	No	PT_2_N_0_M_0_	not available
12	45/F	1.3	Yes	40%	40%CPTC 20%CCVPTC	No	No	Yes(4/4)	No	No	No	PT_1b_N_1_M_0_	73m, NED
13	78/F	2.5	Yes	40%	60%CPTC	No	No	Yes(4/22)	No	No	No	PT_2_N_1_M_0_	74m, NED
14	27/M	3	No	80%	20%CPTC	No	No	Yes(3/9)	No	No	Yes(Bone)	PT_2_N_1_M_1_	53m,AWD
15	65/F	5	No	40%	50%CPTC 10%TCVPTC	Yes	Yes	Yes(2/7)	No	No	No	PT_3_N_1_M_0_	12m,DOC
16	23/M	1.8	No	90%	10% CPTC	No	No	Yes(1/4)	Yes	No	No	PT_1b_N_1_M_0_	68m, NED
17	32/F	1	No	40%	30%CPTC 30%CCVPTC	No	No	Yes(3/12)	Yes	No	No	PT_1a_N_1_M_0_	68m, NED
18	40/F	4	Yes	60%	20%CPTC, 20% FTC	No	No	Yes(3/22)	No	No	No	PT_2_N_1_M_0_	70m, NED

F: female, M: male, LVI: lymphovascular invasion, ETE: extrathyroidal extension, LN: lymph node, METS: metastases, AWD: alive with disease, DOD: died of disease, DOC: died of other causes, NED: no evidence of disease, CPTC: conventional PTC, CCVPTC: Columnar cell-variant papillary thyroid carcinoma, TCVPTC: Tall cell-variant papillary thyroid carcinoma; DSVPTC: Diffuse sclerosing-variant PTC, FVPTC: follicular-variant papillary thyroid cancer

### Sample acquisition

A total of 75 FFPE samples were profiled and only 2 samples from 2 patients (1HVPTC and 1ATC patient) failed sequencing. 73 samples from 55 patients were successfully analyzed, with 29 samples from 17HVPTC patients, 26 samples from 26 CPTC patients, 9 samples from 7PDTC patients and 9 samples from 5 ATC patients. In 16 patients, we obtained more than one FFPE sample from the same patient to investigate the concordance of molecular profiling and possible spatial heterogeneity of these thyroid tumors.

### Quality assessment of the targeted sequencing data

**Figure 2:**
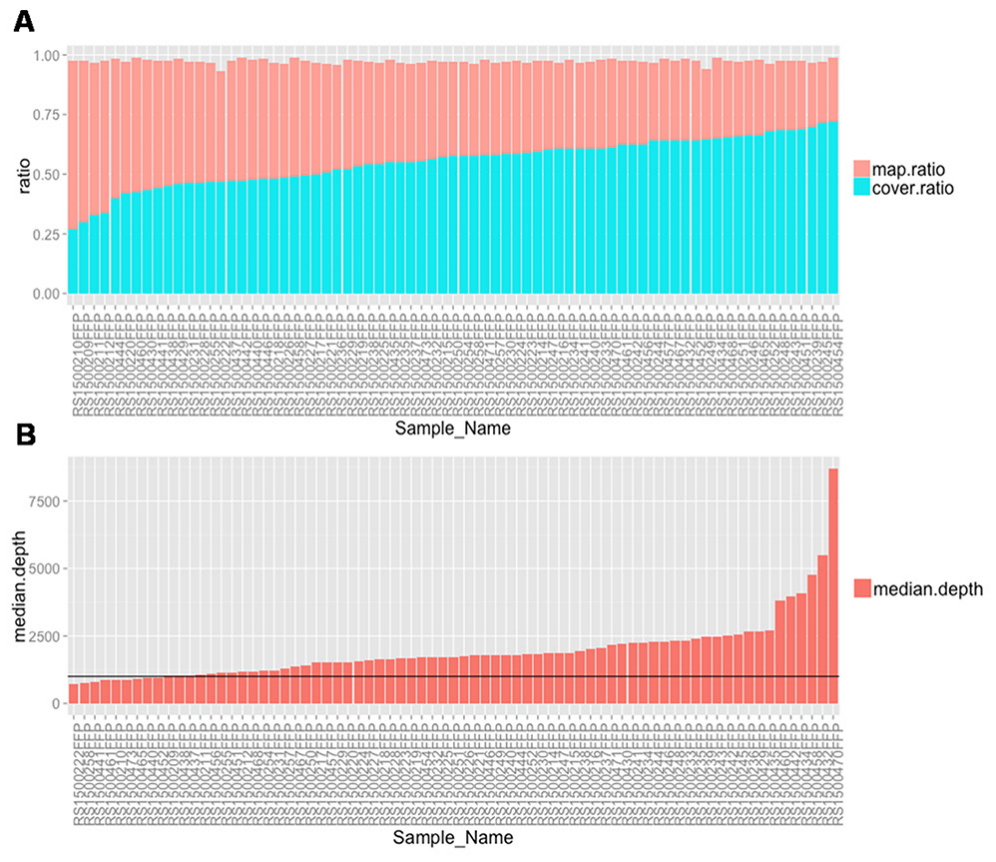
Quality assessment of the targeted sequencing data among 75 samples **(A)** Histogram show the percentage of all mapped reads and percentage of reads mapped to target regions for every sample. **(B)** Bar plot of The median coverage depth among all the target regions for each sample.

Deep sequencing with the 18-gene panel was performed to achieve an average of 1947×coverage among all 73samples profiled. An average of 97.3% of all reads was successfully mapped to the human genome (hg19) and 55.3% of all reads were mapped to our designed target regions (Figure 2A), indicating a high capture efficiency of the designed probes. Figure 2B shows the coverage depth distribution in each sample, where all73 samples have a median coverage depth of more than 500×. Such high coverage enables us to assess mutations present in only a small portion of the tumor cells.

We then assessed the repeatability of probe capturing efficiency by measuring the correlation of coverage depth in each target region between different samples. Supplementary Figure 1A shows a heat map of such correlation between each pair of samples. More than 79.5% of pairs show a statistically significant positive correlation (p<0.05, Pearson correlation), indicating an excellent repeatability of capturing efficiency of probes across different regions. The scatter plots of coverage depth across all regions between the most and least correlated pairs of samples are shown (Supplementary Figure 1B). It can be seen that there is a group of 15 samples (group A) showing weak or even negative correlation with other samples (group B), indicating a different capture enrichment pattern between two types of samples. Supplementary Figure 1C shows the box plots of the imputed DNA fragment length of samples among group A and B. Group A samples have statistically significantly shorter DNA fragments than group B (p<0.0001), indicating a higher level of DNA degradation in group A samples than in group B. Detailed sample characteristics and sequencing data quality assessment parameters are listed out in Supplementary Table 1 and 2.

### Molecular features of HVPTC compared with PTC and ATC/PDTC

**Figure 3:**
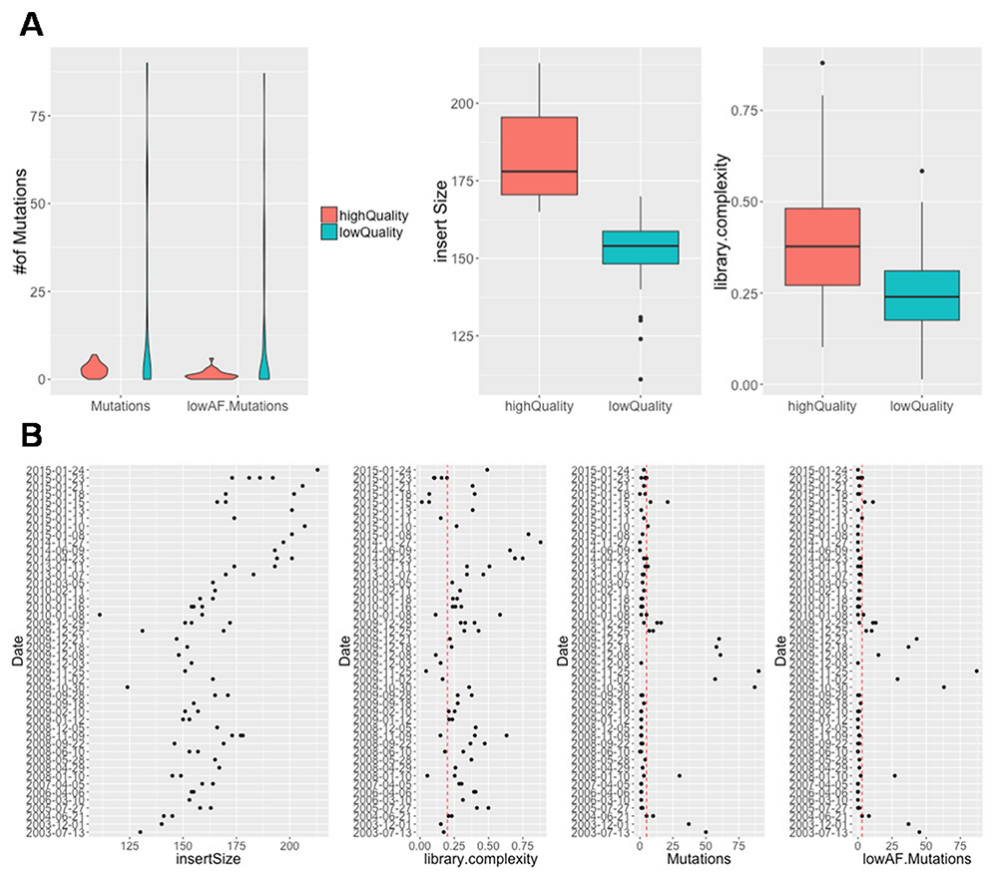
The association of DNA quality and total mutational load among all samples **(A)** Violin plot in left panel show the number of selected mutations and selected low AF mutations between high-quality and low-quality samples. Boxplot in right panel show the insert size and library complexity between high-quality and low-quality samples. **(B)** Correlation between sample collection date and insert size, library complexity, number of mutations, number of low AF mutations illustrated by scatterplot

Targeted sequencing data from all 73 samples were analyzed, and mutations and fusion events were summarized. Since DNA from FFPE samples might degrade over the years, and thus possibly introduce false positive mutations due to DNA damage, we first assessed the DNA quality and associated it with the total mutational load among all samples. Samples with average DNA fragment size lower than 165bp or library complexity lower than 0.1 were defined as low-quality samples and the rest as high-quality samples. Figure 3A shows the box plots of DNA fragment size and library complexity between the two groups, as well as the violin plots of overall mutation number and number of mutations with low allele frequency (AF), which are usually more likely to be caused by DNA damage, between the two groups. It can be seen that both total mutation number and low-AF mutation number are significantly higher among the low-quality samples than high-quality samples (p=0.002, Wilcoxon rank test), as expected. Figure 3B shows the ranked plot of the quality parameters and mutation numbers over the sample collection year. While the DNA fragment size significantly correlates with the collection time (p=4.5e-9, Pearson correlation), indicating a continuous degradation of FFPE DNA over the years, the mutational load is the highest among the oldest samples as well as a group of samples collected from 2009. Given these results, a variant frequency of at least 2% and 10% were used as minimum requirements in this study for high-quality samples and low-quality samples, respectively.

**Figure 4:**
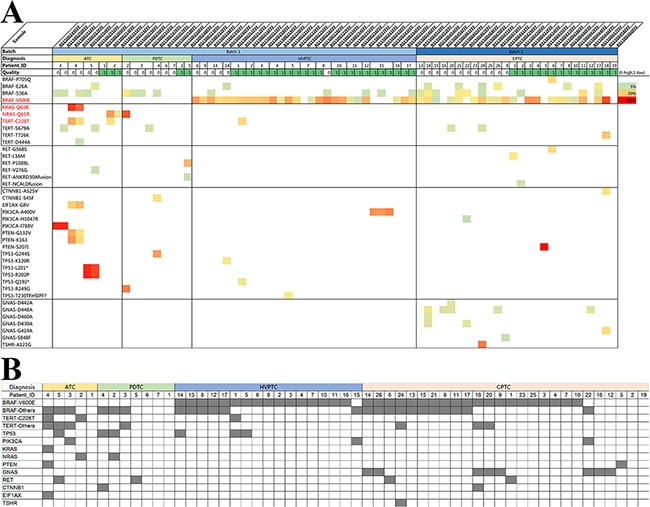
Mutational profiling of HVPTC compared with PTC and ATC/PDTC **(A)** Heatmap of mutations for all samples grouped by subtype and data quality. (mutations are color-coded by AF) **(B)** Representation of the mutation status by Gene-level for all samples grouped by subtype

All mutations identified were summarized in Figure 4A where samples were grouped by subtype, patient, and quality group. Mutations were color-coded by their mutation allele frequency. Mutation status was further combined onto gene level and patient level in Figure 4B. Thirty-seven mutation variant types were identified among 11 genes across all samples. All details about mutations identified can be found in Supplementary Table 3. BRAF V600E mutation was the most common mutation identified, which is present in almost all HVPTC samples (16/17, 94%), most CPTC samples (20/26, 77%), and none of the ATC and PDTC samples. HVPTC harbored an even higher BRAF V600E mutation rate than CPTC, although the difference was not statistically significant.

**Figure 5:**
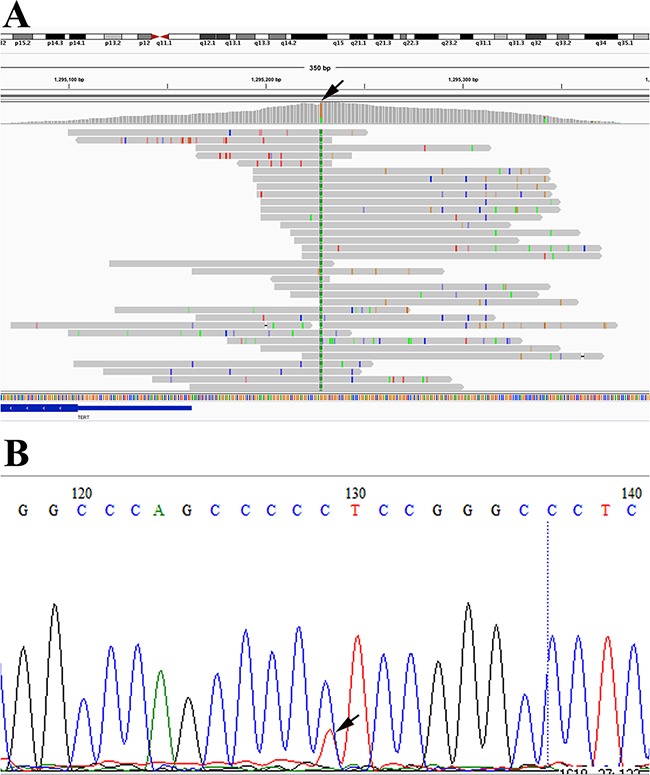
Detection of TERT promoter mutation (C228T) in RS1500212FFP by NGS **(A)** and Sanger sequencing **(B)**, respectively

The promoter mutation variant C228T in TERT, which was recently reported as another distinct biomarker for advanced thyroid cancer, was also identified in 4 samples from 3 patients (2 ATC patients and one HVPTC patient). The TERT promoter mutations were all further confirmed by Sanger sequencing (Figure 5).

RAS family oncogenic mutations and TP53 mutation are almost exclusively present among ATC and PDTC samples. Moreover, TP53 mutation was also observed in 3 HVPTC patients.

Six different GNAS mutations were identified among 8 CPTC patients (31%) and none of the HVPTC patients. There is a statistically significant difference in GNAS mutation rates between these two groups (p=0.01, Fisher exact test). GNAS mutation was also completely absent among all ATC patients and PDTC patients in this study.

We have also investigated the fusion status of RET, ALK, PPARG, and PAX8, and found no fusion events among these samples. Copy numbers of all genes covered in our panel were also imputed and summarized in Supplementary Table 4. No statistically significant copy number variation was identified among any genes in this group of samples.

### Tumor molecular heterogeneity

We next examined the thyroid tumor heterogeneity by comparing mutations identified in different samples from the same patients. Among 16 patients with more than one sample profiled, overall 37 mutation variants were detected. Twenty-one mutations were identified in both samples, while 16 were identified in only one sample. Supplementary Figure 2 shows the scatter plot between the AF of the paired samples from the same patients. It can be seen that the overall AF correlation is rather high between the paired samples (Pearson correlation coefficient = 0.84, p<0.001), indicating great within-patient molecular profile consistency between samples. The vast majority of variants that were only detected in one of the paired samples have an AF lower than 20%.

### Follow-up and molecular features

All patients underwent total or subtotal thyroidectomy with cervical lymph node dissection. Six patients were given ^131^I therapy after surgery. Follow-up data were available for 16 patients with a median of 74 months (range 12-101months). Two patients died during follow-up. However, only one patient experienced local recurrence, lung metastasis and eventually died of disease after 17 months of surgery, whereas the other patient died of angiosarcoma of heart after 5 months of surgery without any thyroid carcinoma recurrence. The patient who died of disease harbored concomitant TERTC228T mutation, BRAF V600E mutation and TP53 mutation. Among 14 patients who remained alive at the last follow-up, one patient who harbored BRAF V600E mutation had bone metastasis after 53 months of surgery and the other 13 patients were without disease recurrence and/or metastases.

## DISCUSSION

Hobnail variant of papillary thyroid carcinoma (HVPTC) was unusual in our PTC cohort, and only 18 out of 1062 (1.69%) cases were diagnosed based on the criteria previously described [3]. So far only 3 study series including more than 10 HVPTC cases have been reported [5, 7, 8]. Hobnail features can also be identified by cytology [14]. Consistent with the previous reports, we observed a relatively large tumor size (mean, 2.53 cm) and high lymph node metastasis rate (58.8 %) with some being multifocal (16.7%) in our HVPTC cases.

The molecular characteristic of HVPTC has been investigated in several recent studies. A high BRAF V600E mutation frequency (74%, range 40-80%) was observed [4, 5]. In the present study, almost all HVPTC cases (16/17, 94.1%) harbored a BRAF V600E mutation, which indicated that BRAF mutation is quite often in HVPTC and might play an important role in its carcinogenesis and cancer progression. As is known, BRAF V600E, a PTC-associated genetic abnormality, is the most prevalent mutation found in CPTC, and has been found to be associated with more aggressiveness and higher risk of recurrence and mortality in a couple of large-cohorts [15, 16]. It is worth noting that, our results reveals that HVPTC harbored an even higher BRAF V600E mutation rate than CPTC, which might partially account for the relatively aggressive biological behavior of HVPTC. However, given that CPTC also harbored a relatively high BRAF V600E mutation rate, BRAF mutation alone might not be sufficient to explain the aggressive behavior of HVPTC.

Recently, two *TERT* promoter mutations, −124 C > T (C228T) and −146 C > T (C250T), have been detected in thyroid cancer cell lines and tissues including well differentiated, poorly differentiated and anaplastic thyroid carcinomas [17–20]. According to previous reports, TERT promoter mutation has been demonstrated to be particularly prevalent in the aggressive thyroid cancers PDTC and ATC [18, 19, 21, 22]. In accordance with these studies, our study also revealed that TERT promoter mutation was mainly present among high-aggressive ATC and PDTC cases but absent from CPTC. So far as we know, only one study particularly investigated the association between TERT promoter mutation and HVPTC, and no TERT promoter mutation was observed in their cohort [8]. Interestingly, TERT promoter mutation was also found in one of our HVPTC patients. Of further note, the HVPTC patient with TERT promoter mutation was seventy-six years old with a relatively large tumor size (5cm) and the only HVPTC patients in our cohort who was died of disease (17 months after surgery). Our results indicated that TERT promoter mutation, as a potential marker of aggressive behavior in thyroid cancers, might also partially account for the relatively aggressive biological behavior of HVPTC. In consistent with our results, previous studies in PTC cohort have also shown a correlation between TERT promoter mutations and older age at diagnosis, larger tumor size, shorter progression free survival and overall survival. All TERT promoter mutations in our cohort were C228T*, and* TERT C250T mutation was not detected, which is consistent with previous reports that C250T mutation was relatively uncommon and mutually exclusive with the C228T in thyroid cancer [18, 22].

It has also been reported that PTCs with BRAF V600E mutation had a higher frequency of TERT promoter mutations than those with wide-type BRAF, and the majority of the TERT promoter mutation-positive PTC samples harbored the BRAF V600E mutation. Moreover, the co-occurrence of TERT promoter mutation and BRAFV600E was associated with the worst clinicopathologic characteristics in classical PTC and the other PTC subtypes as well. Their study demonstrated the patients harboring both BRAF and TERT mutations had an 8.5 fold greater tumor recurrence rate compared with all PTCs patients with neither mutation [23]. However, HVPTC was not included in their study. Interestingly, concomitant BRAF V600E were observed in our HVPTC patient with TERT promoter mutation who died of disease during follow-up, which was consistent with the previous reports that coexistence of BRAF V600E and TERT represented the more aggressive biological behavior in thyroid cancer. However, given the small sample size of our study, the roles of TERT promoter mutation and the correlation between TERT promoter mutation and BRAF V600E mutation in HVPTC needs further clarification in a large cohort.

Besides hobnail variant PTCs, tall cell variant PTCs is commonly recognized as an aggressive subtype of PTCs as well. A couple of studies have confirmed that the prevalence of TERT promoter mutations in tall cell variant PTCs [18, 22], is also much higher than the CPTC cases, and TERT promoter mutation might predict highly significant tumor relapse in tall cell variant PTCs [24]. According to the facts that HVPTCs usually have concomitant tall cell features, and demonstrate similar aggressive characteristics and molecular features to tall cell variant PTCs, a presumption that they belong to the same molecular subtype of PTC might be reasonable but warrant further clarification in large, multicentre studies.

Mutations within TP53 were found in ATC/PDTC but not CPTC samples in our cohort, which is consistent with previous knowledge that oncogenic mutations within these genes correlate with more aggressive cancer subtypes [13, 25]. It is interesting to note that TP53 mutation was also present in 3 of our 17 HVPTC cases, and one HVPTC case with TP53 mutation was the above-mentioned patient with concurrent BRAF V600E and TERT C228T mutations. Our results suggested that TP53 mutation might also contribute to the relatively aggressive behavior of HVPTC. However, the correlation between TP53 mutation and BRAF or TERT mutation in thyroid cancer remains unclear. In fact, McFadden DG et al has demonstrated P53 loss enabled progression from PTC to ATC in a BRAF-mutant mouse model of papillary thyroid cancer [26].

A significant association between mutant RAS and more aggressive behavior of follicular and papillary carcinoma has been reported, which may be due to the role of RAS mutation in promoting tumor dedifferentiation and transformation to anaplastic carcinoma [27]. In consistent with this, we detected RAS mutations in three PTC/ATC cases (one KAS mutation and two NRAS mutations). However, no RAS mutations were observed in our HVPTC cases, which indicated RAS mutations might have little significance in HVPTC. Nonetheless, given the small sample size, the conclusions should be regarded with caution.

GNAS mutation was considered a marker of a benign or well-differentiated disease in the previous reports, including McCune-Albright syndrome [28], thyroid toxic nodules [29], pituitary adenoma [30], and ovarian granulosa cell tumor [30, 31]. A significant frequency of GNAS mutations in CPTC (8/26) was an unexpected finding in our study. Moreover, GNAS mutations were only found in CPTC but not in HVPTC and ATC/PDTC, which also supported that there might be obvious molecular differences between CPTC and HVPTC. However, the results need validation in a larger series.

RET/PTC1 gene rearrangement has been reported in HVPTC cases by previous studies [5, 32]. However, gene fusion and copy number alterations were not observed in any of our thyroid cancer cases. Given the limitations of genomic structural variation detection in long-term archived FFPE tissue by targeted NGS, the results should be interpreted with caution.

Intratumor molecular heterogeneity has been observed in this cohort and indicated that heterogeneity does exist in thyroid tumors. There are mutations that might only be carried by a small proportion of the tumor cells, and thus their mutation status could differ among different tissue samples of the same patient. These mutations can only be detected and studied by highly sensitive techniques, such as NGS.

In summary, HVPTC is a rare and aggressive variant of PTC. The molecular mechanisms underlying its aggressive behavior remain unclear. So far as we know, our study is the first to explore the genetic changes of HVPTC using targeted NGS-based 18-gene panel. In this study, the molecular features of HVPTC were investigated in comparison with, CPTC and ATC/PDTC using NGS. BRAF V600E mutation, which is the most prevalent mutation in CPTC and indicates more aggressiveness, was found to be even higher in HVPTC but not present in ATC/PDTC. TERT promoter mutation, another potential marker of aggressive behavior in thyroid cancers, was also observed in HVPTC but not in CPTC, and was concurrent with BRAF V600E mutation. Moreover, TP53 mutation was also present in HVPTC patients but not in CPTC. Finally, neither RAS nor GNAS mutation was found in HVPTC. Our results suggested that as an aggressive variant of PTC, HVPTC has relatively specific molecular features, which is somewhat different from both CPTC and ATC/PDTC and may underlie its relatively aggressive behavior. Nonetheless, given the small sample size and the limitation of our single-center study, the conclusions warrant further clarification in large, multicentre, prospective studies.

## MATERIALS AND METHODS

### Case selection

A total of 1062 consecutive cases diagnosed as PTC and treated at the Peking Union Medical College Hospital (PUMCH) were collected between 2000 and 2010. All the slides were submitted to re-review by two pathologists with special expertise in thyroid tumor (T.L and L.Z). A total of 18 HVPTC cases were diagnosed using the following criteria: 1). Growth pattern predominantly papillary or papillary-follicular, with predominance of micropapillary structures in some cases; 2). ≤10% of the tumor showing tall cell/columnar pattern or diffuse sclerosing patterns; and 3). ≥30% tumor cells harboring hobnail features, which are characterized by cuboidal cells with high nuclear cytoplasmic ratio and apically placed nuclei with bulging of the apical surface [3]. In addition, 26 CPTCs, 7 PDTCs and 6 ATCs diagnosed during the same period were also included in this study.

### Thyroid sample preparation

An appropriate paraffin block containing tumor tissue was selected for analysis after the haematoxylineosin (H&E)-stained slides reviewed by an experienced pathologist. Tumor area on the H&E-stained slide was marked and manually micro-dissected from up to 10 unstained sections to enrich for tumor cells before DNA extraction. A minimum of 30% tumor cell content was required in the present study

### DNA isolation and library preparation

DNA of FFPE samples was extracted (QIAamp DNA FFPE tissue kit; QIAGEN, Valencia, CA) and the DNA concentration was measured by QubitdsDNA assay. The gDNA quality was assessed to make sure that A260/A280 is within the range of 1.8 to 2.0. Shearing fragmentation by sonication (covaris M220;Covaris, Inc., USA) was conducted, followed by end repair, phosphorylation and adaptor ligation. Fragments of size 200-400bp were selected by bead (AgencourtAMPure XP Kit; Agilent Technologies, Palo Alto, CA), followed by hybridization with the capture probes baits, hybrid selection with magnetic beads, and PCR amplification. A bioanalyzer high sensitivity DNA assay was then performed to assess the quality and size of the fragments. All indexed samples were then sequenced on NextSeq500 (Illumina, Inc., USA) with pair-end reads.

### Targeted DNA panel design

The capture probe baits were designed to cover 140kb human genomic loci from 186 target regions, including selected exons and introns from 18 genes (BRAF, NRAS, HRAS, KRAS, RET, NTRK1, ETV6, ALK, PPARG, TERT, EIF1AX, PTEN, AKT1, PIK3CA, TP53, CTNNB1, TSHR, GNAS) [12], so that we were able to detect all point mutation, indel, and CNV events as well as fusion events of important genes with any partner. The SureSelect reagents were prepared using the Agilent eArray platform and the probes were manufactured by Agilent.

### Sequencing data analysis

Sequencing data were mapped to the human genome (hg19) using BWA aligner 0.7.10. PCR duplicate reads were removed before base substitution detection. Local alignment optimization and variant calling was performed using GATK v3.2-2. DNA translocation analysis was performed using both Tophat2 and Factera 1.4.3. Insert size distribution and library complexity of each sample were computed to assess the level of DNA degradation. Different mutation calling thresholds were applied on samples with different DNA quality to avoid false positive mutation calls due to DNA damage. SNV and indels identified were annotated using the dbNSFP(v30a), COSMIC (v69), and dbSNP (snp138)database. Variants with a global minor allele frequency greater than 1.0% in 1000Genome Project (Phase3,http://www.1000genomes.org/data) were considered as common SNPs, and removed. Integrative Genomics Viewer (Broad Institute, USA) was used to visualize variants aligned against the reference genome to confirm the accuracy of the variant calls by checking for possible strand biases and sequencing errors. Copy number variation (CNV) analysis was performed by normalizing and read counts from each target region and the gene-level CNV was assessed by a z-test.

## SUPPLEMENTARY MATERIALS FIGURES AND TABLES









## References

[1] Chen W, Zheng R, Baade PD, Zhang S, Zeng H, Bray F, Jemal A, Yu XQ, He J (2016). Cancer statistics in China, 2015. CA Cancer J Clin.

[2] Motosugi U, Murata S, Nagata K, Yasuda M, Shimizu M (2009). Thyroid papillary carcinoma with micropapillary and hobnail growth pattern: a histological variant with intermediate malignancy?. Thyroid.

[3] Asioli S, Erickson LA, Sebo TJ, Zhang J, Jin L, Thompson GB, Lloyd RV (2010). Papillary thyroid carcinoma with prominent hobnail features: a new aggressive variant of moderately differentiated papillary carcinoma. A clinicopathologic, immunohistochemical, and molecular study of eight cases. The American journal of surgical pathology.

[4] Amacher AM, Goyal B, Lewis JS, El-Mofty SK, Chernock RD (2015). Prevalence of a hobnail pattern in papillary, poorly differentiated, and anaplastic thyroid carcinoma: a possible manifestation of high-grade transformation. The American journal of surgical pathology.

[5] Lubitz CC, Economopoulos KP, Pawlak AC, Lynch K, Dias-Santagata D, Faquin WC, Sadow PM (2014). Hobnail variant of papillary thyroid carcinoma: an institutional case series and molecular profile. Thyroid.

[6] Lino-Silva LS, Dominguez-Malagon HR, Caro-Sanchez CH, Salcedo-Hernandez RA (2012). Thyroid gland papillary carcinomas with “micropapillary pattern”, a recently recognized poor prognostic finding: clinicopathologic and survival analysis of 7 cases. Human pathology.

[7] Asioli S, Erickson LA, Righi A, Lloyd RV (2013). Papillary thyroid carcinoma with hobnail features: histopathologic criteria to predict aggressive behavior. Human pathology.

[8] Lee YS, Kim Y, Jeon S, Bae JS, Jung SL, Jung CK (2015). Cytologic, clinicopathologic, and molecular features of papillary thyroid carcinoma with prominent hobnail features: 10 case reports and systematic literature review. International journal of clinical and experimental pathology.

[9] Metzker ML (2010). Sequencing technologies - the next generation. Nature reviews Genetics.

[10] el M Bahassi, Stambrook PJ (2014). Next-generation sequencing technologies: breaking the sound barrier of human genetics. Mutagenesis.

[11] Nikiforov YE, Carty SE, Chiosea SI, Coyne C, Duvvuri U, Ferris RL, Gooding WE, LeBeau SO, Ohori NP, Seethala RR, Tublin ME, Yip L, Nikiforova MN (2015). Impact of the Multi-Gene ThyroSeq Next-Generation Sequencing Assay on Cancer Diagnosis in Thyroid Nodules with Atypia of Undetermined Significance/Follicular Lesion of Undetermined Significance Cytology. Thyroid.

[12] Nikiforov YE, Carty SE, Chiosea SI, Coyne C, Duvvuri U, Ferris RL, Gooding WE, Hodak SP, LeBeau SO, Ohori NP, Seethala RR, Tublin ME, Yip L (2014). Highly accurate diagnosis of cancer in thyroid nodules with follicular neoplasm/suspicious for a follicular neoplasm cytology by ThyroSeq v2 next-generation sequencing assay. Cancer.

[13] Nikiforova MN, Wald AI, Roy S, Durso MB, Nikiforov YE (2013). Targeted next-generation sequencing panel (ThyroSeq) for detection of mutations in thyroid cancer. The Journal of clinical endocrinology and metabolism.

[14] Schwock J, Desai G, Devon KM, Mete O, Dube V (2015). Hobnail-variant of papillary thyroid carcinoma in liquid-based cytology. Diagnostic cytopathology.

[15] Li C, Lee KC, Schneider EB, Zeiger MA (2012). BRAF V600E mutation and its association with clinicopathological features of papillary thyroid cancer: a meta-analysis. The Journal of clinical endocrinology and metabolism.

[16] Xing M, Alzahrani AS, Carson KA, Viola D, Elisei R, Bendlova B, Yip L, Mian C, Vianello F, Tuttle RM, Robenshtok E, Fagin JA, Puxeddu E (2013). Association between BRAF V600E mutation and mortality in patients with papillary thyroid cancer. Jama.

[17] Landa I, Ganly I, Chan TA, Mitsutake N, Matsuse M, Ibrahimpasic T, Ghossein RA, Fagin JA (2013). Frequent somatic TERT promoter mutations in thyroid cancer: higher prevalence in advanced forms of the disease. The Journal of clinical endocrinology and metabolism.

[18] Liu X, Bishop J, Shan Y, Pai S, Liu D, Murugan AK, Sun H, El-Naggar AK, Xing M (2013). Highly prevalent TERT promoter mutations in aggressive thyroid cancers. Endocrine-related cancer.

[19] Melo M, AG da Rocha, Vinagre J, Batista R, Peixoto J, Tavares C, Celestino R, Almeida A, Salgado C, Eloy C, Castro P, Prazeres H, Lima J (2014). TERT promoter mutations are a major indicator of poor outcome in differentiated thyroid carcinomas. The Journal of clinical endocrinology and metabolism.

[20] de Biase D, Gandolfi G, Ragazzi M, Eszlinger M, Sancisi V, Gugnoni M, Visani M, Pession A, Casadei G, Durante C, Costante G, Bruno R, Torlontano M (2015). TERT Promoter Mutations in Papillary Thyroid Microcarcinomas. Thyroid.

[21] Liu T, Wang N, Cao J, Sofiadis A, Dinets A, Zedenius J, Larsson C, Xu D (2014). The age- and shorter telomere-dependent TERT promoter mutation in follicular thyroid cell-derived carcinomas. Oncogene.

[22] Qasem EY, Murugan AK, Al-Hindi HS, Xing M, Al-Mohanna M, Alswailem M, Alzahrani AS (2015). TERT promoter mutations in thyroid cancer: a report from a Middle Eastern population. Endocrine-related cancer.

[23] Xing M, Liu R, Liu X, Murugan AK, Zhu G, Zeiger MA, Pai S, Bishop J (2014). BRAF V600E and TERT promoter mutations cooperatively identify the most aggressive papillary thyroid cancer with highest recurrence. Journal of clinical oncology.

[24] Dettmer MS, Schmitt A, Steinert H, Capper D, Moch H, Komminoth P, Perren A (2015). Tall cell papillary thyroid carcinoma: new diagnostic criteria and mutations in BRAF and TERT. Endocrine-related cancer.

[25] Sykorova V, Dvorakova S, Vcelak J, Vaclavikova E, Halkova T, Kodetova D, Lastuvka P, Betka J, Vlcek P, Reboun M, Katra R, Bendlova B (2015). Search for new genetic biomarkers in poorly differentiated and anaplastic thyroid carcinomas using next generation sequencing. Anticancer research.

[26] McFadden DG, Vernon A, Santiago PM, Martinez-McFaline R, Bhutkar A, Crowley DM, McMahon M, Sadow PM, Jacks T (2014). p53 constrains progression to anaplastic thyroid carcinoma in a Braf-mutant mouse model of papillary thyroid cancer.

[27] Nikiforov YE (2011). Molecular analysis of thyroid tumors. Modern pathology.

[28] Weinstein LS, Shenker A, Gejman PV, Merino MJ, Friedman E, Spiegel AM (1991). Activating mutations of the stimulatory G protein in the McCune-Albright syndrome. The New England journal of medicine.

[29] Nishihara E, Amino N, Maekawa K, Yoshida H, Ito M, Kubota S, Fukata S, Miyauchi A (2009). Prevalence of TSH receptor and Gsalpha mutations in 45 autonomously functioning thyroid nodules in Japan. Endocrine journal.

[30] Tordjman K, Stern N, Ouaknine G, Yossiphov Y, Razon N, Nordenskjold M, Friedman E (1993). Activating mutations of the Gs alpha-gene in nonfunctioning pituitary tumors. The Journal of clinical endocrinology and metabolism.

[31] Kalfa N, Ecochard A, Patte C, Duvillard P, Audran F, Pienkowski C, Thibaud E, Brauner R, Lecointre C, Plantaz D, Guedj AM, Paris F, Baldet P (2006). Activating mutations of the stimulatory g protein in juvenile ovarian granulosa cell tumors: a new prognostic factor?. The Journal of clinical endocrinology and metabolism.

[32] Ieni A, Barresi V, Cardia R, Licata L, Di Bari F, Benvenga S, Tuccari G (2016). The micropapillary/hobnail variant of papillary thyroid carcinoma: A review of series described in the literature compared to a series from one southern Italy pathology institution. Reviews in endocrine & metabolic disorders.

