# Supplementary material for: Hobnail variant of papillary thyroid carcinoma: molecular profiling and comparison to classical papillary thyroid carcinoma, poorly differentiated thyroid carcinoma and anaplastic thyroid carcinoma
Source: Oncotarget. 2017 Feb 28;8(13):22023–33. doi: 10.18632/oncotarget.15786 (PMC5400643; doi:10.18632/oncotarget.15786)
Supplement: Supplementary file 1 [file oncotarget-08-22023-s001.pdf]

Hobnail variant of papillary thyroid carcinoma: molecular profiling and comparison to classical papillary thyroid carcinoma, poorly differentiated thyroid carcinoma and anaplastic thyroid carcinoma

SUPPLEMENTARY FIGURES AND TABLES

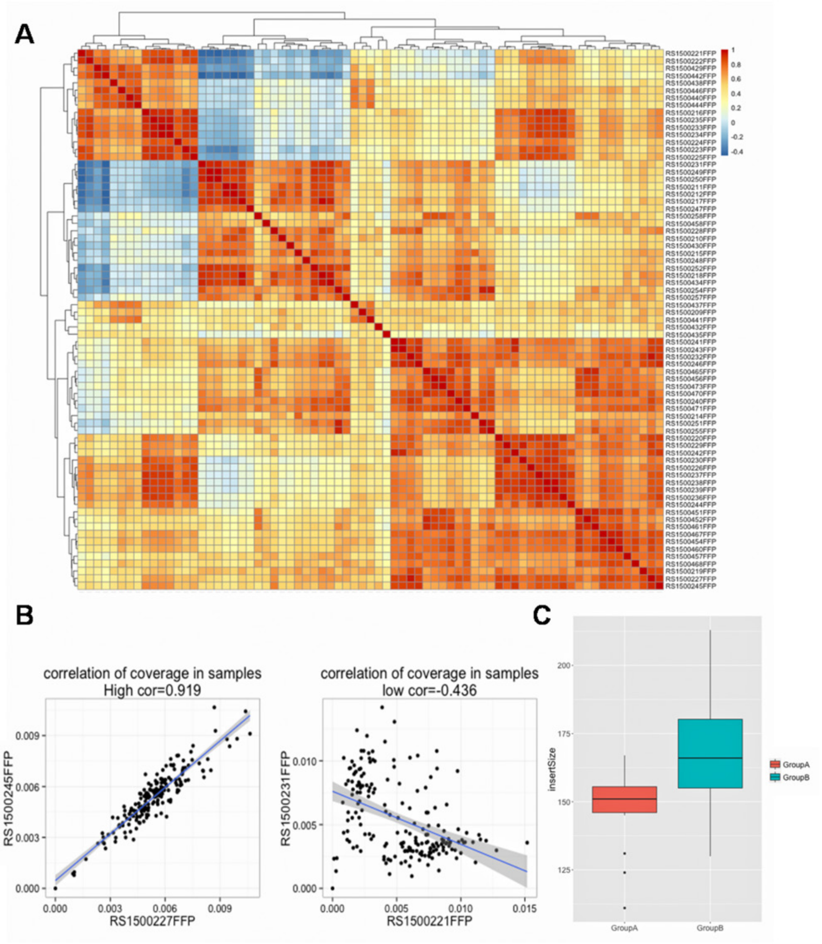

Supplementary Figure 1: The correlation of coverage depth in each target region between samples. **(A)** A heatmap of coverage depth correlation between samples. **(B)** The scatterplot of coverage depth across all regions in the positive correlated pair of samples. **(C)** The scatterplot of coverage depth across all regions in the negative correlated pair of samples. Boxplot show the insert size between two groups classified by pair-wise correlation. (group A are the 15 samples in top-left of Figure 2A, others are group B).

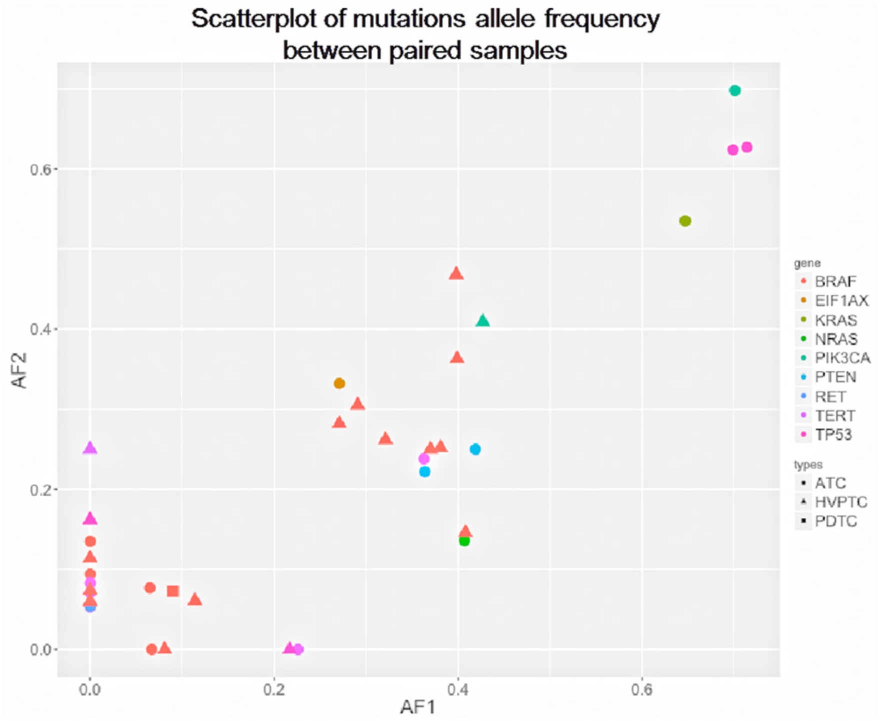

Supplementary Figure 2: Scatterplot show the mutations AFs between the paired samples from same patients. Each mutation point is colored by gene and shaped by subtype.

Supplementary Table 1: Clinical information of all patient samples.

Supplementary File 1

Supplementary Table 2: Quality assessment of the targeted sequencing data.

Supplementary File 2

Supplementary Table 3: Detail mutation list of all samples.

Supplementary File 3

Supplementary Table 4: Copy numbers of genes in panel for all samples.

Supplementary File 4
